# A study on the embarrassment of senders who missend emojis with opposite meanings on social apps: taking WeChat as an example

**DOI:** 10.1186/s41155-020-00159-4

**Published:** 2020-08-24

**Authors:** Liyuan Liu, Yen Hsu, Jie Zhang, Qianling Jiang

**Affiliations:** 1 College of Science & Technology, Ningbo University, Ningbo, China; 2 The Graduate Institute of Design Science, Tatung University, Taipei, Taiwan; 3 School of Design, The Hong Kong Polytechnic University, Hong Kong, China; 4 School of Design, Jiangnan University, Wuxi, China

**Keywords:** WeChat, Emoji with opposite meaning, Missending, Sender, Embarrassment, Interaction design

## Abstract

With the increasing popularity of social apps, sending emojis has become a very common way of expressing one’s emotions. However, situations often arise when people send the wrong emoji by mistake, or sometimes even an emoji with an opposite meaning, which can cause embarrassment to the sender. Taking WeChat as an example, which is widely used in Chinese communities, this study summarizes 10 types of dialogue situations in which the meaning of an emoji is wrongly sent and 12 types of emotional components that are related to embarrassment. The purpose of this study was to analyze the extent to which the 12 emotional components that are associated with embarrassment actually explain what embarrassment is, as well as the different degrees of embarrassment among the different genders and age groups. The results showed that (1) among the emotional components of embarrassment, shame has the highest explanation degree for embarrassment; (2) males are more likely to be affected by embarrassment than females; and (3) users aged 18–25 and 26–30 years are more likely to be affected by embarrassment than those aged between 31 and 40 when they mistakenly send WeChat emojis. This study provides a reference value for their sustainable psychological impact on social app users.

## Introduction

Amidst the fierce competition of mobile real-time social software, it is becoming more important to maintain the users’ willingness to continue using the software (Zhang et al., 2017). Mobile Instant Messengers (MIMs), such as Messenger, WhatsApp, LINE, and WeChat, are very popular, and sending emojis has become a very common way of expressing one’s emotions (Annamalai & Salam, 2017). Researchers have found that while emojis are very popular, the wrong ones or even those with an opposite meaning are sometimes mistakenly sent by users, when one is not expecting to do so; it may cause embarrassing emotions.

Contact and communication with others are important activities in the lives of people, and information transmission can be facilitated by means of WhatsApp, WeChat, LINE, and Telegram. In China, WeChat has dramatically changed peoples’ social lifestyles, and contact has become an indispensable part of their daily lives (Danesi, 2016). According to data from Tencent in March 2018, WeChat has more than 1 billion active users worldwide every day. WeChat is the most important mobile MIMs in the Chinese-speaking world (Wang et al., 2019). According to a survey, around 25% of the users open WeChat over 30 times per day, and 55.2% use WeChat over 10 times per day (Wen et al., 2016). However, most past studies have only focused on Facebook, YouTube, or other international MIMs that are most popular in Western countries (Montag et al., 2018; Tsai & Men, 2018). Few studies have focused on the beneficial effects of Chinese MIMs on its users, especially WeChat (Research, 2016).

Pang (2018a, 2018b) showed that there are four factors that describe a person’s willingness to use WeChat, namely, time, emotion, social interaction, and fashion. Research shows that 69.4% of users use WeChat daily; 12.2% use it three to four times a week; and 11.6% use it five to six times a week. Although the frequency of the use of WeChat per week can be better predicted by changes in the time spent (e.g., whether it was for pleasant rest, leisure, and relaxation, or for helping and thanking others) (Zhang et al., 2017), the main motivation of 84.4% of the users was to have an emotional connection with everyone they knew (Hossain et al., 2019). The interaction between complex emotions and ambiguous information comprehension makes the information more ambiguous and further affects the cognition of emojis (Zhang et al., 2017).

It is very common and popular to communicate by using emojis. From smartphones to social media, small digital graphics are ubiquitous and people are very willing to send these graphics (Pang, 2018a). The study showed that 90% of the people found communicating with emojis more meaningful (Pang, 2016). Emojis not only serve as a user’s emotional guide, but also provide many other communicative functions (Riordan, 2017a). Although there are enough graphics available for downloading, each graphic might not be used for a long time, and some downloaded emojis will be discarded, but built-in emojis will be kept and continue to be used (Tseng & Hsieh, 2019).

The symbol for a smiling face as an emoticon is “^_^”, while as an emoji, it is a small symbol represented by a picture, such as the symbol for a smiling face “ ”. Emojis are used in almost the same way as emoticons (Jaeger et al., 2018), but it does not mean that they are used with the same frequency, or that they have the same influence. Previous studies have shown that Twitter users using emojis have reduced their use of emoticons because emojis are replacing emoticons in fulfilling the same paralinguistic functions (Danesi, 2016). It has been noted that the large number of emojis that describe a wide range of content has reduced the need for the use of emoticons (Pavalanathan & Eisenstein, 2016) and that emojis are now more commonly used than emoticons (68.1% vs 30.9%) (Li & Yang, 2018). Although digital dialogues are mainly by means of written communication, non-verbal communication, such as via emojis, can facilitate emotional expression and improve the efficiency of dialogues (Chen et al., 2017), and 11.3% of people use emojis to shorten the time it takes to send a message (Ganster et al., 2012).

This study conducted psychological research on the emotional communication function of WeChat emojis, for the following reasons: (1) emojis are used by almost all social media users to start and maintain conversations and to facilitate interpersonal relationships (Wang et al., 2019); (2) during network-based communications, the use of emojis exhibits a high frequency, function, and efficiency (Choi et al., 2017; Hanna et al., 2017; Lomanowska & Guitton, 2016; Yang & Lee, 2020); (3) the communication function of emojis, therefore, enhances the emotional relationship between people (Dhir & Tsai, 2017; Pang, 2018b); and (4) during emotional communication in the social environment, people often use positive emojis to create a positive atmosphere and to promote harmonious relations (Kelly & Watts, 2015). However, misunderstanding the meaning of an emoji may cause communication barriers, and in specific situations, it may damage relationships (Li & Yang, 2018). As there are only a few research studies on the psychological effects of WeChat on users (Danesi, 2016; Kaye et al., 2016), this paper looks to fill a gap in the related literature through an analysis of missent emojis.

Previous studies have emphasized the functional role, the condition for use, and the usefulness of emojis in facilitating communication (Li & Yang, 2018). However, few studies have looked at the impact on the emotions of the sender of missending WeChat emojis with an opposite meaning. The emotional role of an emoji is expounded in sociological theory, which notes that the time and effort spent on the use of emojis can help to maintain and strengthen social relations (Tigwell & Flatla, 2016).

Embarrassment is an important emotion that affects social communication, and it plays a very important role in maintaining and developing social relations (Pang, 2018a). Research has found that embarrassment will lead to negative consequences (Castillo et al., 2013; Derks et al., 2007; Pauleen & Yoong, 2001; Tseng & Hsieh, 2019). Previous research by Riordan (2017b) has pointed out that embarrassment is a combination of such emotions as bewilderment, clumsiness, shame, and annoyance. The negative consequences of embarrassment will lead to a decline in the users’ satisfaction with WeChat, and this will have an impact on their willingness to continue using WeChat (Bastin et al., 2016). WeChat users have complained on social media, that they have sent wrong emojis because the WeChat emojis that have opposite meanings are arranged close to each other. The contribution of this study is that it not only has theoretical significance in network social psychology, but it also has a practical significance for improving the WeChat users’ willingness to continue using WeChat.

However, we know little about the differences between the emoji usage of men and women (McCambridge & Consedine, 2014) and that different emojis are used by different ages (Miller, 1996). In order to make up for the lack of studies on the differences in emoji usage among users of different genders and ages and to improve the continuous willingness of users to use WeChat emoji, the main purposes of this study are as follows:

1. To explore which gender is more easily affected by embarrassment after missending a WeChat emoji with an opposite meaning;
2. To explore which age group is more easily affected by embarrassment after missending a WeChat emoji with an opposite meaning; and
3. To analyze the senders’ embarrassment after missending WeChat emojis, in order to determine the emotional components that are related to embarrassment and their degree of influence.

This study can help to promote people’s willingness to continue using WeChat and to provide a theoretical basis for developing a good social networking situation. The findings of this study will provide a reference for promoting positive emotions in future MIMs.

## Methods

### Participants

The Internal Review Board of the Neural Ergonomics Laboratory has approved this study. This study adopts the online questionnaire survey format. All participants click the webpage link of the survey to view the survey description of the study; they voluntarily answer the research questions and can quit the survey at any time. Therefore, all participants agreed to participate in the study under the principle of fully informed and voluntary participation.

To study the link between gender differences and embarrassment, this study treated 30 men and 30 women as the subjects.

Our study surveyed participants in 10 conversation scenarios involving relationships between family members, relatives, lovers, friends, and leaders. In order to meet the requirements of the sample in this study, it is necessary for participants to master all the interpersonal relationships in these 10 dialogues. Therefore, from the 378 samples, we selected a number of samples that met the research criteria and which includes all the interpersonal experiences in 10 conversation scenarios.

After this step, our selected sample covered interpersonal relationships in 10 conversation scenarios, which met the needs of the study. However, not every participant met this requirement. Most participants had difficulty matching the interpersonal relationships in all the conversation scenarios. Of the 378 samples, only a few matched the interpersonal relationships covered by the 10 conversation scenarios.

In a sample of 378 questionnaires, we first selected a sample of 30 women who exactly matched the interpersonal relationships contained in the 10 conversation scenarios. In psychological research, the common practice of defining a sample is usually to include the same number of men and women in a single sample (Sigel, 1965). In order to keep the sample size of men and women equal, we conducted a second round of questionnaire screening, by selecting a sample of 30 men, which exactly matched the interpersonal relationships contained in the 10 conversation scenarios. Therefore, the final sample of this study was 30 men and 30 women, which was consistent with the number of sample plans in this study.

### Procedure and materials

This study is divided into four stages, namely (1) the emoji layout of WeChat and the determination of their opposite meanings; (2) the collection and analysis of dialogue scenarios that were caused by mistakenly sending WeChat emojis with opposite meanings; (3) the identification of the emotional components related to embarrassment; and (4) the preparation of questionnaires and the respondents answering the questions by selecting the intensity of the emotional components, according to the dialogue scenarios.

#### Phase 1—the emoji layout of WeChat and the determination of its opposite meaning

**Fig. 1:**
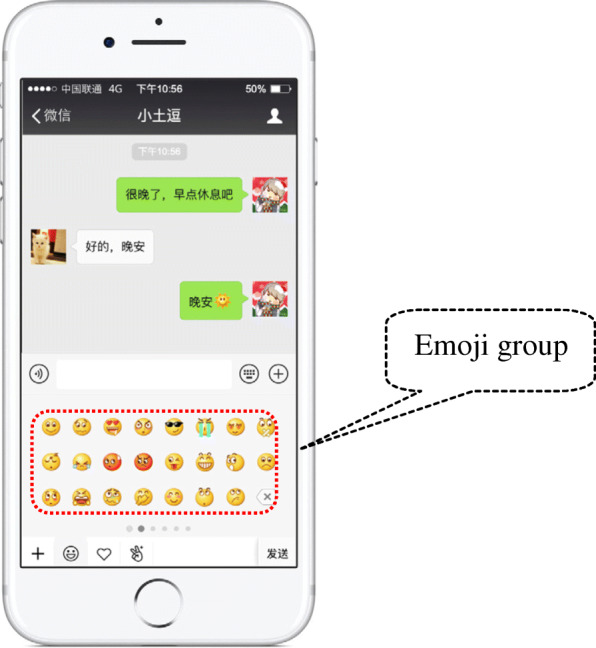
The WeChat (Version: 6.7.4) interface and its field of emojis

**Fig. 2:**
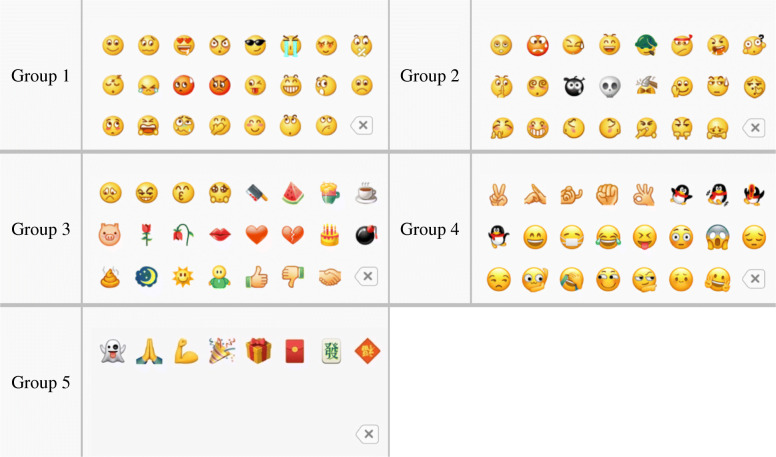
WeChat’s built-in basic emoji groups

The dialogue interface for sending a WeChat emoji is shown in Fig. 1. The area that is surrounded by the red dotted line is the emoji group. Figure 2 shows the layout of all the emojis of WeChat. There are 104 built-in basic emojis in WeChat, which are divided into five groups (Fig. 2), and they can be selected by sliding the cursor left and right. Groups One, Two, Three, and Four have three lines each, with 23 emojis in each group. The last position of the last line of each group (the lower right-hand corner of the emoji group) is occupied by the deletion symbol. Group Five only has one line and is comprised of eight emojis.

Based on the perspective of the emoji sender, this study determined the opposite meaning of an emoji from a specific graphic meaning, context, and culture. (1) In WeChat, every emoji has a specific meaning. Pressing the emoji for a long time will prompt the meaning of the emoji. Users send the emojis based on what they mean, such as “  ” for strong and “  ” for weak. By knowing the specific meanings of emojis, users can clearly determine their opposite meanings, without ambiguity and polysemy. (2) When actually using the emojis, their meanings are very contextual, sometimes with complex, multi-layered or overlapping meanings (Darics, 2013). For example, “  ” can mean not only surprise and confusion, but also doubt (Tannen, 1994). Therefore, the context is very important for understanding emoticons and determining their opposite meanings. (3) Emojis have different meanings in different cultures (Ljubešić & Fišer, 2016). Emoticons reveal cultural diversity (Freedman, 2018). For example, “  ” has five main meanings in the world, with “good” being the dominant interpretation (Morris, 1979), but the “thumbs up” in Iraq or in Sweden is an insult and means “up yours”. The respondents in this study are Chinese WeChat users who have a common cultural background, so they have a relatively consistent understanding of the opposite meanings of WeChat emojis.

Emojis with opposite meanings were collected from all dialogue situations, including two types, namely (1) the dialogue situation captured in WeChat screenshots and (2) the dialogue situation described by the sender through a text. According to the specific meaning, context, and culture of emojis, we analyzed their opposite meanings in the dialogue situations that were collected. We extracted two emojis with opposite meanings in each dialogue situation and took the two emojis with opposite meanings in each situation as a pair. Through the analysis of the layout position of emojis with opposite meanings in the dialogue situation, we found that in different contexts, users mistakenly sent emojis with opposite meanings. This occurred mainly between two adjacent emojis, including the upper and lower adjacent emojis and the left and right adjacent emojis. As Groups One, Two, and Three have three lines each, there may be cases of missending two horizontally or vertically adjacent emojis, and because Group Five has only one line, the case of missending vertically adjacent emojis does not occur.

**Fig. 3:**
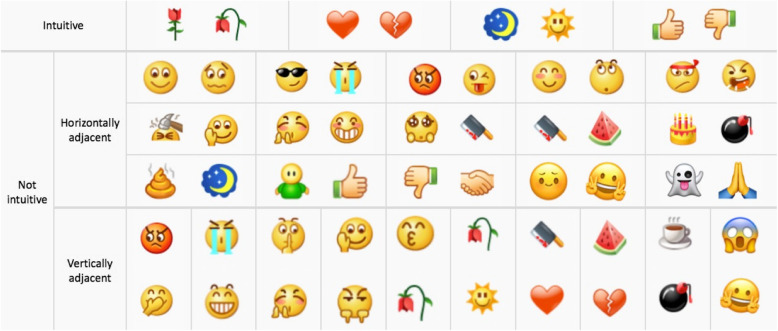
Classification of WeChat emojis with opposite meanings

There are two kinds of emojis with opposite meanings that may be mistakenly sent by users. The one is when the two adjacent emojis have obviously opposite meanings in visual form, i.e., they are intuitive and easily identifiable emoji graphics, including four pairs, and the other is that the opposite meanings of two emoji graphics are not intuitive in visual form, but will cause opposite results according to the dialogue situation (Tseng & Hsieh, 2019). There are 25 pairs. In summary, there are 29 pairs of emojis with opposite meanings in WeChat, as can be seen in Fig. 3.

#### Phase 2—the selection and analysis of situations

The collection of dialogue scenarios on embarrassing topics is very sensitive as it involves interpersonal relationships and personal emotions (Bethell et al., 2014; Uysal et al., 2014). There is sufficient evidence to show that the transmission of information is usually related to emotions and identities (Bas-Hoogendam et al., 2018; Hofmann et al., 2006; Sharkey & Singelis, 1995). In this study, the anonymity of the interviewees is the key factor, especially regarding the integrity and accuracy of the collection of dialogue scenarios. Therefore, the dialogue scenarios are collected by anonymous online questionnaires. There are two main forms of respondent feedback on the dialogue scenarios, namely (1) the respondents who described, in writing, about the dialogues in which they had sent WeChat emojis with opposite meanings by mistake; and (2) the respondents took screenshots of the dialogues in which they had sent WeChat emojis with opposite meanings by mistake. In total, 103 anonymous online questionnaires were collected with conversational scenarios that involved the missending of WeChat emojis with opposite meanings.

In the 103 conversations, the more frequently a pair of emojis with opposite meanings appeared indicated that the pair was more likely to be confused by the sender. In the conversation situations that we collected, the opposite emojis with “  ” appeared the most, 14 times in total, with a relative frequency of 13.59%. Compared with other emojis with opposite meanings, emojis are the most likely to be confused by the sender. Emojis “  ”, “  ”, “  ” and “  ” also appear with relatively high frequency, suggesting that these emojis are also easily confused by the sender.

Based on the 103 collected conversations, we studied emojis and contexts as a whole and took the seven intentions for sending emojis (Hu et al., 2017) as a reference index to analyze the different psychological states and frequency of sending emojis with opposite meanings. The results are shown in Table 1. Emojis with opposite meanings have the highest frequency of expressing emotions (34.95%), while those with opposite meanings have a higher frequency of strengthening the expression (22%). The top 10 most-used emojis are mostly positive, while the bottom 10 are mostly negative. The smiley emoji “  ” was the most used (63%) (Lin, 2019).

**Table 1:** Psychological state of the sender when sending emojis

	Frequency	Relative frequency
Emotional expression	36	34.95%
Strengthen the expression	22	21.36%
Adjust the mood	10	9.71%
Express a sense of humor	5	4.85%
Express satire	0	0.00%
Express intimacy	8	7.77%
Describing the content	9	8.74%
Others and unknown	13	12.62%
Total	103	100%

We also measured how often emojis with opposite meanings appeared in different relationships. An analysis of 103 conversation scenarios found that the interpersonal relationships in which the expressions with opposite meanings were incorrect, included friends, relatives, colleagues, lovers, and leaders, which are common in network communication (Hongqiang et al., 2019).

As shown in Table 2, the frequency of sending emojis with opposite meanings is the highest among friends (30.10%). The second highest is the relative frequency between relatives (18.45%), and the lowest frequency was for the leadership category, with a relative frequency of 8.74%. This could be explained by the fact that emojis are used more in intimate relationships and less when communicating with people who are in a distant relationship. In task-oriented formal communication, in order to avoid communication difficulties caused by the different interpretation of emojis, people may prefer to use meaningful words to clearly convey the information (Hongqiang et al., 2019).

**Table 2:** The frequency of emojis with opposite meanings in different relationships

Relationships	Frequency	Relative frequency
Friends	31	30.10%
Relatives	19	18.45%
Colleagues	15	14.56%
Lovers	10	9.71%
Leaders	9	8.74%
Others and unknowns	17	16.50%
Total	103	100%

The location of the WeChat emojis in context is divided into four categories, namely, the single, beginning, middle, and end. WeChat emojis are rarely seen in the beginning and the middle. Emojis were not used in combination with other words, and the relative frequency with which they appeared separately was 34.2%, while the relative frequency with which they appeared at the end was the highest (51.7%) (Hongqiang et al., 2019). This is consistent with the position of emojis that were collected in the dialogue scenes of this study, while the positions of emojis with opposite meanings sent by the sender appeared mainly at the end of the dialogue.

Three linguistic experts were invited as judges to analyze and classify the themes of the 103 conversations, according to the relationship between the sender and receiver and to summarize the dialogue scenarios with a similar content. The top 10 situations with the most similar topics and those that occurred most frequently were selected (see Appendix). The 10 dialogue scenarios contained common interpersonal relationships and were representative in dialogues where the missending of emojis with opposite meanings existed. For example, the user originally wanted to send “  ”, indicating a compliment, but actually mistakenly sent “  ”, indicating the opposite meaning. Table 3 lists three dialogue scenarios to exemplify the occurrence of embarrassment.

**Table 3:** Examples of dialogue scenarios involving the missending of WeChat emojis

No.	Right	Opposite (Missent)	Example
1			The mother told the father that “our son got first place in the competition today”. The father wanted to send to show his compliment to the son’s performance, but mistakenly sent with the opposite meaning, resulting in embarrassment.
2			A couple was sending messages to each other. The girl texted, “Keep warm. Miss you”. The boyfriend wanted to send “OK, I love you ”, but mistakenly sent “OK, I love you ”, which led to the embarrassment of the boyfriend.
3			A friend asked “Are you free tonight? Welcome to my birthday party.” The receiver wanted to reply with the graphic of a birthday cake to express the best wishes for the birthday, but mistakenly sent “ ”, resulting in embarrassment.

In order to protect the privacy of the interlocutors and to avoid the influence of the personal profile photos, names, and nicknames of the respondents, their personal information, such as profile photos, names, or nicknames, in the dialogue scenarios were reprocessed. The content and explanations of the 10 dialogue scenarios are listed in the Appendix below.

#### Phase3—determination of the emotional components

A consideration concerns the construct that psychological tests purport to measure (Miguel & Pessotto, 2016). Through a literature review and analysis, this study determined the main sources of the following 12 emotional components that are related to embarrassment: (1) the following eight embarrassment-related emotional components are proposed by Grace (2007), namely, “Angry”, “Humiliated”, “Upset”, “Self-conscious”, “Foolish”, “Frustrated”, “Depressed”, and “Shocked” and (2) the four emotional components related to embarrassment are proposed by other literature, namely, “Distressed”, “Fearful”, “Anxious”, and “Ashamed”. These are shown in Table 4.

**Table 4:** Twelve embarrassment-related emotional components

No.	Emotional components of embarrassment	References
1	Angry	(Bethell et al., 2014)
2	Humiliated	(Bethell et al., 2014; Uysal et al., 2014)
3	Upset	(Bastin et al., 2016; Higuchi & Fukada, 2002; Kaufman, 2004; Lewis, 1971; Modigliani, 1971)
4	Self-conscious	(Grace, 2007)
5	Foolish	(Grace, 2007)
6	Frustrated	(Babcock, 1988; Grace, 2007; Miller, 1996; Parrott & Smith, 1991)
7	Depressed	(Grace, 2007)
8	Shocked	(Grace, 2007)
9	Distressed	(Babcock, 1988; Grace, 2007; Miller, 1996; Parrott & Smith, 1991)
10	Fearful	(Grace, 2007)
11	Anxious	(Grace, 2007)
12	Shame	(Bethell et al., 2014)

#### Phase 4—questionnaire design and survey

In order to avoid the situation where participants focus only on answering the first few questions and then possibly weaken their answers to the subsequent questions about embarrassing emotions, the questionnaire randomly arranged the words for the 12 emotional components of embarrassment, or that are related to embarrassment, to ensure the quality of the questionnaire responses. According to the degree, from strong to weak, the respondents chose the strength of each word to describe the situation. The questionnaire of this study adopted the Likert 7-point scale to divide the level of strength of the emotional components that are related to embarrassment. The questionnaire data were collected in an anonymous manner by a professional online research company (https://www.sojump.com/), which has rich experience in working in China (Borg et al., 1988; Higuchi & Fukada, 2002; Kaufman, 2004; Lewis, 1971; Modigliani, 1971). By using the network’s questionnaire survey, we actually collected a total of 378 samples.

### Statistical analysis

The total reliability coefficient of the data is 0.945, which indicates that the reliability of the research data is very high.

For the research data, we conducted the following: (1) a regression analysis to analyze the impact of 12 embarrassment-related emotional components on embarrassment and (2) a two-way ANOVA to analyze the relationship between independent variables (gender and age) and embarrassment.

## Results

Through experiments and analysis, it was found that (1) the average value of embarrassment and its emotional components for males is significantly higher than that for females; (2) compared to users between 31 and 40 years old, users aged between 18 and 5 years are more susceptible to embarrassment when they mistakenly send WeChat emojis, while users aged between 26 and 30 years are also susceptible to embarrassment; and (3) the degree of shame caused by embarrassment is the highest.

### Gender and embarrassment

**Fig. 4:**
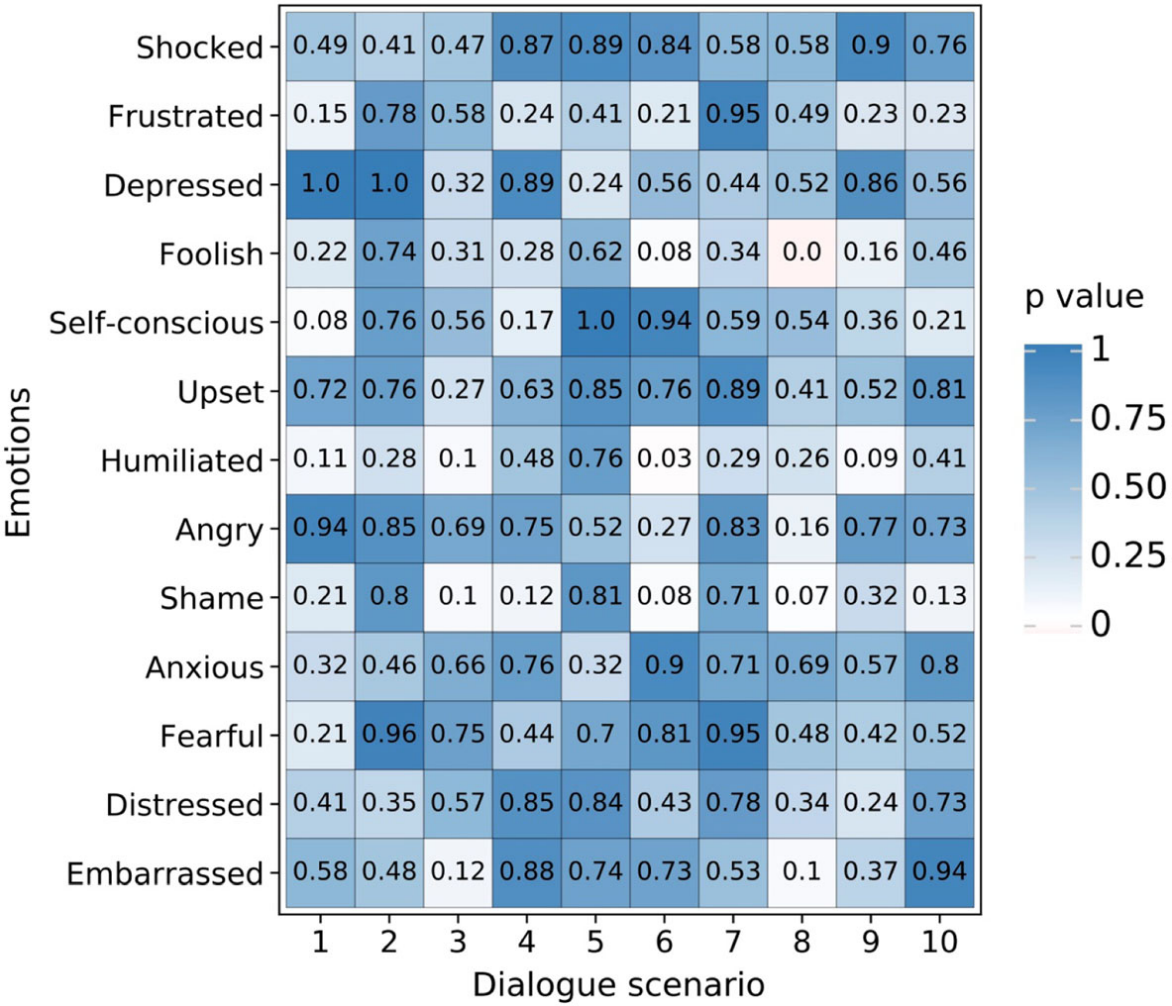
The two-way ANOVA thermogram of gender and embarrassment components

From the questionnaires on the relationship between gender and embarrassment components, a statistical analysis was carried out on the questionnaires of the 30 male and female participants, respectively. The analysis was presented visually by using data analysis technology by Python software, as shown in Fig. 4.

The explanation of Fig. 4 is as follows: the *X*-axis shows the 10 dialogue scenarios that were used to test embarrassment; the *Y*-axis is embarrassment and its emotional components; the color blocks represent the *P* values, ranging from 0 to 1, from white to blue. The two-way ANOVA was used to study the different gender aspects in 1 + 12 items, namely, Embarrassed, Shocked, Frustrated, Depressed, Foolish, Self-conscious, Upset, Humiliated, Angry, Ashamed, Anxious, Fearful, and Distressed. From the two-way ANOVA thermogram, the following can be seen: in Scenario 6, gender is significant in Humiliated, which indicates that the subjects of different genders exhibit differences in being Humiliated; in Scenario 8, gender is significant in Foolish, which indicates that the subjects of different genders show differences in feeling Foolish; except for the above differences, different genders do not show differences in feeling Embarrassed, Shocked, Frustrated, Depressed, Foolish, Self-conscious, Upset, Humiliated, Angry, Ashamed, Anxious, Fearful, and Distressed (*P* > 0.05), which indicates that subjects of different genders are relatively consistent and have no differences in the above emotions.

**Fig. 5:**
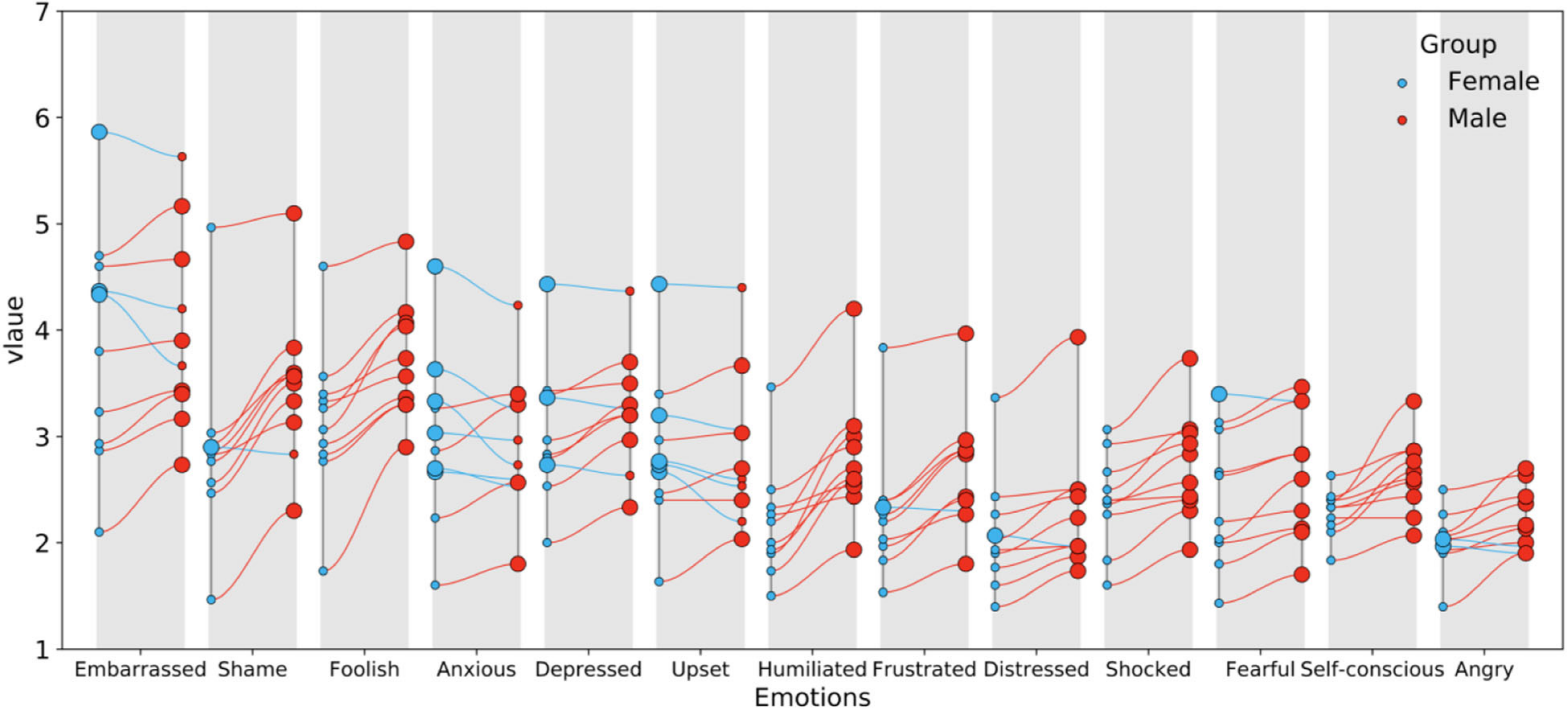
Mean values of females and males for different emotions under 10 dialogue scenarios

Although the smaller the *P* value, the more significant the statistical test is, it is difficult to quantify the *P* value in the probability scale as the data strength for the nihility hypothesis. Even if the null hypothesis is rejected, there is still a high probability (about 20%) that null is true (Grace, 2007). Therefore, we adopted the mean values of female and male for different emotions under 10 dialogue scenarios, graphed them in Fig. 5 by using data analysis technology and the function of data visualization by Python, and then conducted a further analysis.

The explanation of Fig. 5 is as follows: the *X*-axis represents embarrassment and its emotional components; and the numbers 2, 3, 4, 5, and 6 on the *Y*-axis represent the scale scores. When the average score of males is higher than that of females in the same scenario, the red solid line is used to show a connection; a larger red circle is used to represent the score value of the males; when the average score of the females is higher than that of males in the same scenario, the blue solid line is used for a connection; and the larger blue circle is used to represent the score value of the females. Figure 5 shows that there are obviously more red lines than there are blue lines. Therefore, the average value of embarrassment and emotional components of males is significantly higher than that of the females.

### Age and embarrassment

**Fig. 6:**
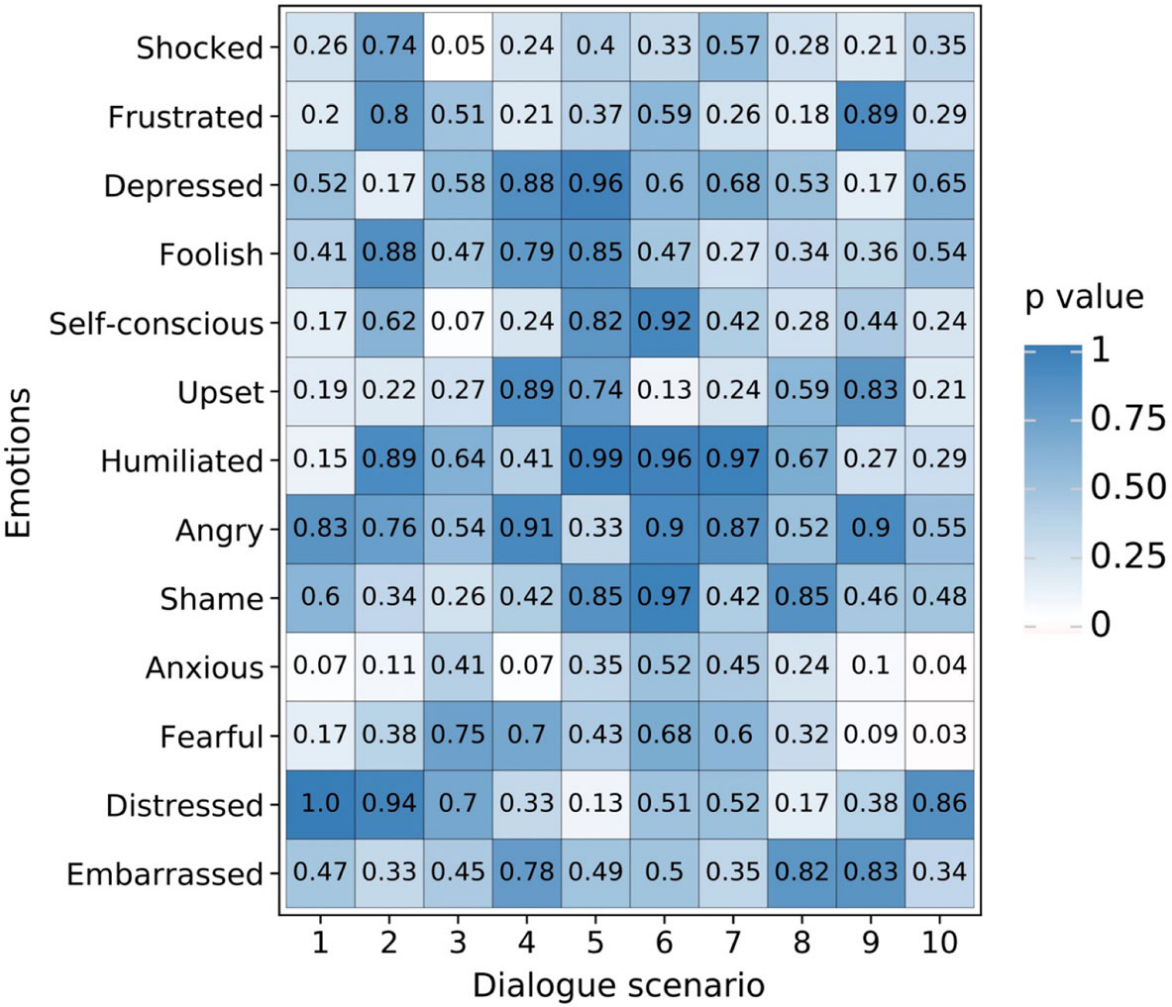
A two-way ANOVA thermogram of age and embarrassment components

The explanation of Fig. 6 is as mentioned in the previous section. The two-way ANOVA was used to study the differences of 13 items, including Shocked, Frustrated, Depressed, Foolish, Self-conscious, Upset, Humiliated, Angry, Ashamed, Anxious, Fearful, Distressed, and Embarrassed. The two-way ANOVA thermogram shows the following: in Scenario 10, different ages show significance in Anxious and Fearful, which indicates that the subjects of different ages exhibit differences in Anxiety and Fearfulness; except for the above differences, subjects of different ages do not show any significance in Shocked, Frustrated, Depressed, Foolish, Self-conscious, Upset, Humiliated, Angry, Shame, Anxious, Fearful, Distressed, and Embarrassment, which indicates that the subjects of different ages are consistent and that there are no significant differences in Embarrassment, Shocked, Frustrated, Depressed, Foolish, Self-conscious, Upset, Humiliated, Angry, Shame, Anxious, Fearful, and Distressed.

**Fig. 7:**
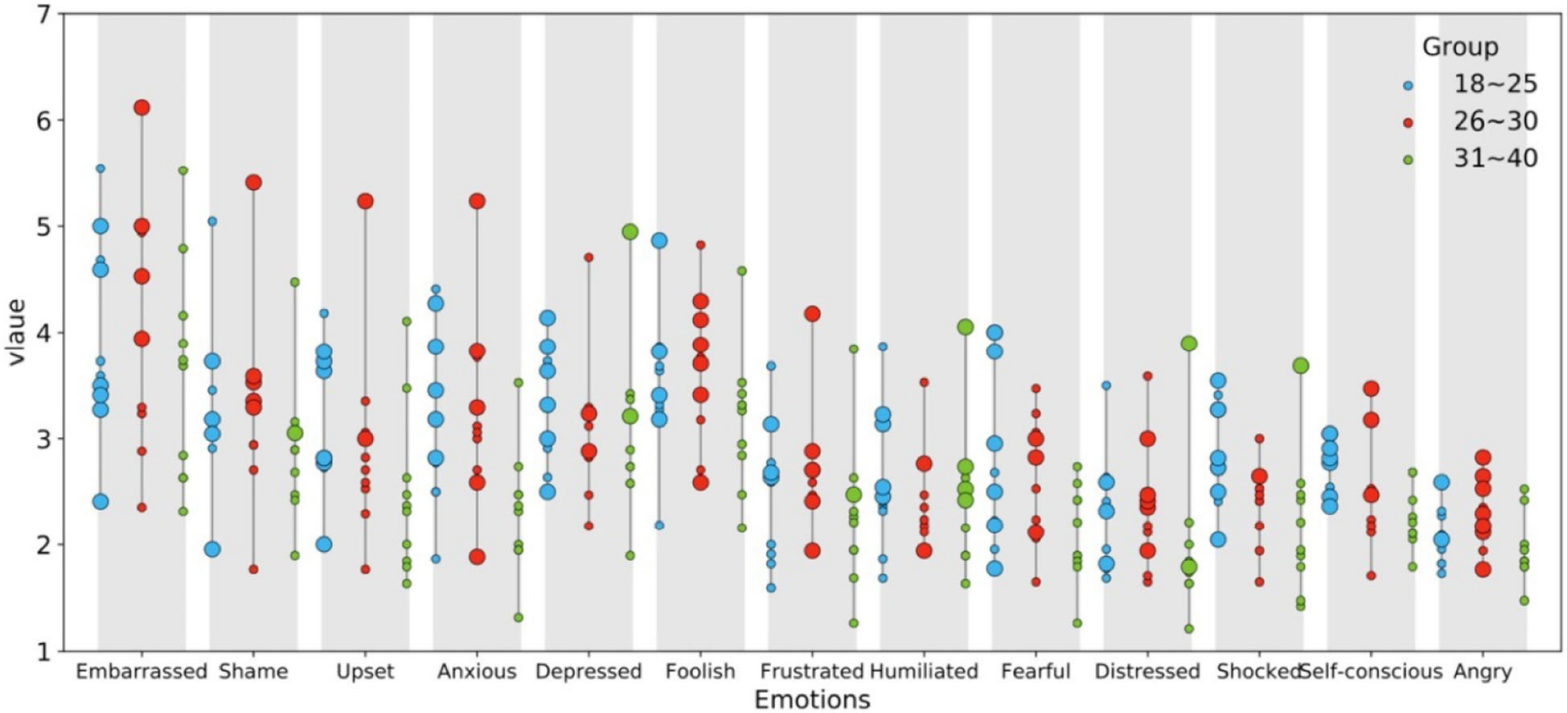
The mean value of respondents of three age groups for different emotions under 10 dialogue scenarios

Combining the description about the *P* value in the previous section, we adopted the mean value of the respondents with the three age groups for different emotions under 10 dialogue scenarios. Figure 7 is plotted by using data analysis technology and the function of data visualization by Python software and conducted further analysis.

The explanation of Fig. 7 is as follows: the *X*-axis represents embarrassment and its emotional components; the *Y*-axis represents the scale scores; when the average score of an age group is higher than that of the other two age groups in the same scenario, it is represented by a larger circle. The 31–40-year age group has small green circles in all scenarios, indicating that it has lower scores, and the average scores for Embarrassment and emotional components are obviously lower than those for the 18–25-year-old and 26–30-year-old age groups. These age groups are basically consistent in showing larger blue and red circles in each scenario, which indicates that the emotional evaluations of both groups are consistent. In summary, the average score of age groups in Embarrassment or certain emotional components is “18–25-years olds ≈ 26–30-year olds > 31–40-year olds”.

### Regression analysis

**Fig. 8:**
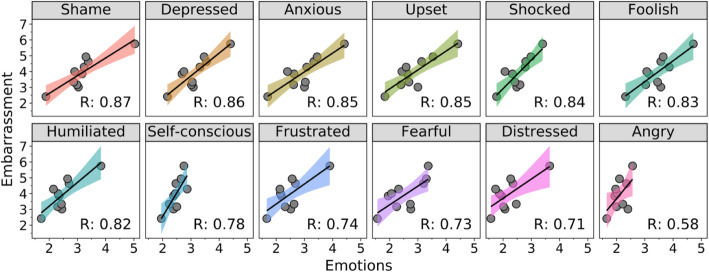
Relationship between embarrassment and emotions

As shown in Fig. 8, the regression analysis was made by taking Shame, Depressed, Anxious, Upset, Shocked, Foolish, Humiliated, Self-conscious, Frustrated, Fearful, Distressed, and Angry as the independent variables and Embarrassment as the dependent variable. Among them, the degree of explanation of Shame for Embarrassment is 0.87, that of Depressed for Embarrassment is 0.86, that of Anxious and Upset for Embarrassment is 0.85, that of Shocked for Embarrassment is 0.84, that of Foolish for Embarrassment is 0.83, and that of Humiliated for Embarrassment is 0.82, while the degrees of explanation of Self-conscious, Frustrated, Fearful, and Distressed for Embarrassment are between 0.78 and 0.71, at 0.78, 0.74, 0.73, and 0.71, respectively. The degree of explanation of Angry for Embarrassment is 0.58, which is the lowest and thus has a comparatively lower explanation for Embarrassment, compared with other emotions.

**Fig. 9:**
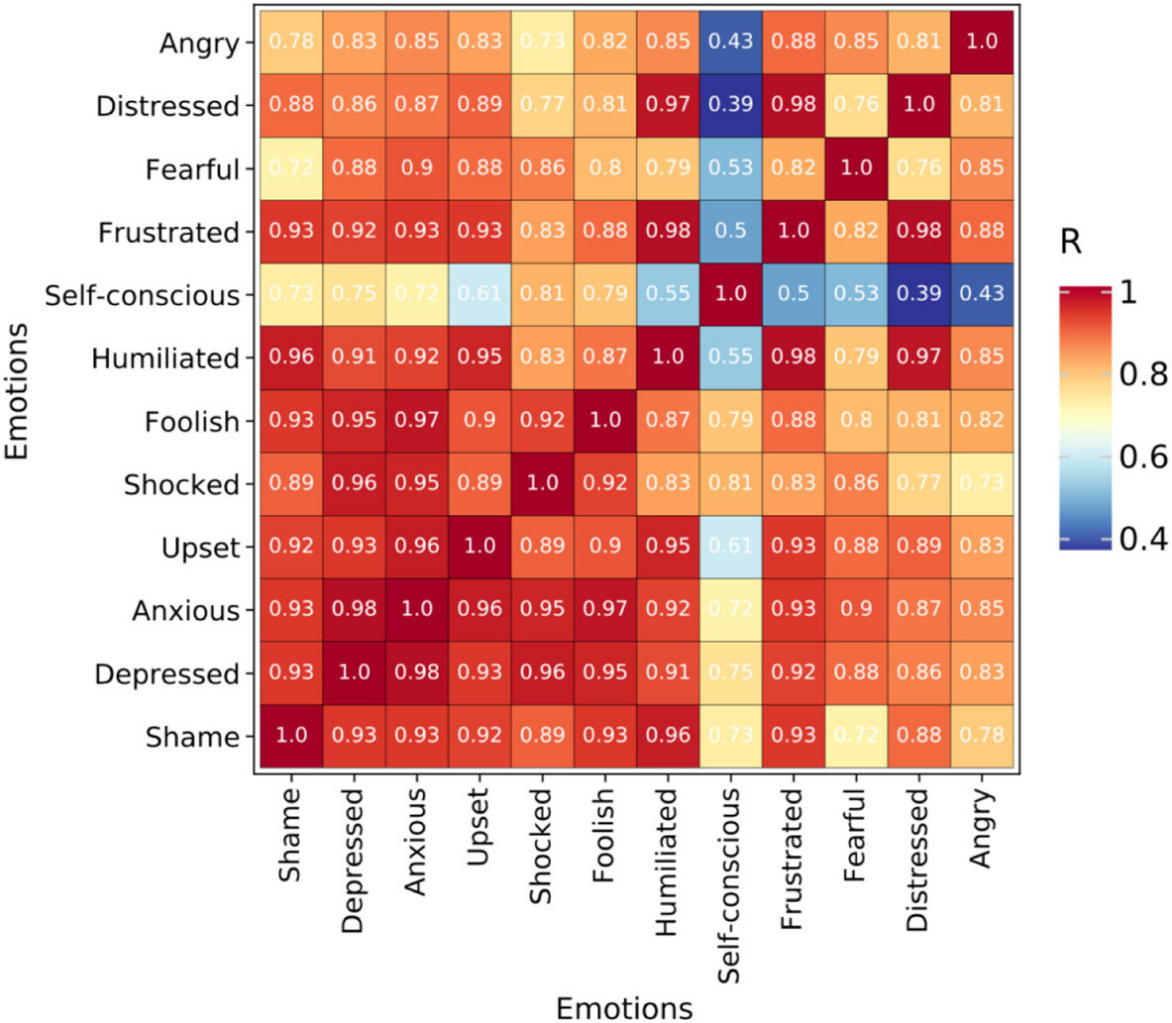
Thermodynamic diagram of correlation coefficients

This study presented a thermodynamic diagram of correlation coefficients by using data analysis technology and the function of data visualization by Python software, as shown in Fig. 9. It examined 12 emotions, namely, Angry, Distressed, Fearful, Frustrated, Self-conscious, Humiliated, Foolish, Shocked, Upset, Anxious, Depressed, and Shame. The blocks of different colors indicate the strength of the correlation; red indicates the high correlation between the two emotions; blue indicates the low correlation between the two emotions; and yellow indicates the moderate correlation between the two emotions. As can be seen in Fig. 9, most of the color blocks are red, which implies that the correlation is generally high. The 11 emotions of Angry, Distressed, Fearful, Frustrated, Humiliated, Foolish, Shocked, Upset, Anxious, Depressed, and Shame have a correlation coefficient of more than 0.7, which shows a high correlation. The correlation coefficients of Self-conscious and other emotions are generally low, of which the lowest correlation coefficient is for Distressed, which is 0.39 and marked in dark blue, followed by Angry, Frustrated, Fearful, Humiliated, and Upset, with correlation coefficients of 0.43, 0.5, 0.53, 0.55, and 0.61, respectively, and which are marked with blue blocks. The values of all correlation coefficients are greater than 0, which indicate positive correlations—that is to say, the corresponding two emotions will strengthen or weaken at the same time.

In view of the analysis results of Fig. 9, the traditional multivariate linear regression cannot be used for an accurate calculation, due to the multicollinearity between the independent variables of the emotions. Therefore, this study adopted the regression algorithm of LASSO (least absolute shrinkage and selection operator), which can improve the interpretability and accuracy of the model (Song et al., 2016). The regression equation of Embarrassment is

$$ \mathrm{Embarrassment}=0.687\mathrm{Shame}+0.108\mathrm{Anxious}+0.226\mathrm{Upset} $$

Here, the best alpha is 0.081, and R^2^ is 0.758.

## Discussion

This study takes sent WeChat emoji with opposite meanings as an example for discussing the embarrassment of the sender. “Embarrassment” is a situation that cannot be avoided in interpersonal relationships; it reflects a person’s feeling of the appropriateness of behavior, causes a person’s frustration, and forms a negative emotion (Babcock, 1988). In this study, the degrees of explanation of shame for Depressed, Anxious, Upset, Shocked, Foolish, and Humiliated for Embarrassment are all above 0.82, which is high.

Shame has the highest explanation for Embarrassment, which is consistent with the opinion of Borg et al. (1988). Embarrassment is always considered as a dimension of Shame (Oleszkiewicz et al., 2017). In other languages of modern society, Embarrassment is regarded as a minor version of Shame. For example, “verguenza” in Spanish has two meanings: embarrassment and shame. In many languages, the commonly used word “shame” is also a kind of embarrassment (Evans, 2015).

Scholars have pointed out that the anxiety is an important feature of embarrassment (Jankowski & Takahashi, 2014). Among general people, higher levels of anxiety are associated with greater embarrassment (Bas-Hoogendam et al., 2018), and social anxiety is closely related to embarrassment (Hofmann et al., 2006). Previous research (Miller, 1996) has defined embarrassment more clearly as a self-perceptible emotion that has a blurring line with anxiety and shame.

“Embarrassment” makes people feel constrained, which takes a period of time to recover from. Embarrassment also destroys a pleasant mood. Once embarrassment occurs, people enter a state of panic, clumsiness, shame, and chagrin, which makes them feel very uncomfortable (Miller, 1996). Fifty percent of the people with embarrassed feelings will also feel humiliation (Grace, 2007).

When users mistakenly send WeChat emojis with an opposite meaning, it can cause embarrassment and have a negative impact on relationships, but sometimes it cannot be said to be harmful. It is an emotional response to the sudden occurrence of seemingly harmless, and sometimes humorous, events (Miller, 1996). Embarrassment makes the individual feel stupid (Goffman, 1967).

The Embarrassment of mistakenly sending an emoji with opposite meanings involves self-esteem. People with different degrees of self-esteem react differently to embarrassment (Song et al., 2017), which is caused by the potential loss of self-esteem (Modigliani, 1971), including the negative evaluation of others (Modigliani, 1971), one’s own behavior not conforming to certain ideal traits (Babcock, 1988), the failure to present a consistent image (Parrott & Smith, 1991), a violation of other people’s expectations, etc. (Borg et al., 1988).

“Embarrassment” makes people feel constrained, which takes a period of time to recover from. Embarrassment also destroys a pleasant mood. Once embarrassment occurs, people enter a state of panic, clumsiness, shame, and chagrin, which makes them feel very uncomfortable (Miller, 1996). This study explains the embarrassing psychological processes and emotional components of the WeChat emoji with opposite meanings that were mistakenly sent.

People usually want to leave a good impression in their social circle, but when they make careless mistakes while attending activities, they will feel embarrassed and ashamed (Kaufman, 2004; Lewis, 1971), as was the case of the emotional state of the sender in this study after sending WeChat emoji incorrectly.

These findings highlighted that the key components are Shame, Depressed, Anxious, Upset, Shocked, Foolish, and Humiliated, which deserve enough attention and play an important role in improving the users’ willingness to continue using WeChat. In particular, reducing the users’ emotional reactions of shame helps to improve the users’ willingness to continue using WeChat.

Compared with the other embarrassment of components discussed above, namely, Self-conscious, Frustrated, Fearful, Distressed and Anger, this explains that the degree of embarrassment is low. According to previous research on embarrassment that is related to consumption, anger accounts for the lowest proportion of embarrassing emotions. In studies on the embarrassing emotional reactions caused by missending emojis, as compared with other emotions, anger can less likely explain the change of embarrassment. Research has produced different results, which represents the complexity of emotions (Borg et al., 1988). It also shows that in WeChat’s emoji, which mistakenly sends the opposite meaning, anger can explain embarrassment, but does not bring a strong angry embarrassment to the sender.

In this study, although gender and age had no significant influence on embarrassment, a further analysis showed that people of different genders and ages were affected to different degrees by embarrassment.

When sending WeChat emoji with opposite meanings, males are more easily affected by embarrassment, compared with females. This can be explained by the fact that although females use more emojis than males (Wu et al., 2019), they are stronger than men in enduring certain emotional stress. Males will be significantly affected by psychological factors, while females will not, and their strength continues to grow (Lin & Yin, 2015). The results of this study show that men were more embarrassed than women when they mistakenly sent an emoji with an opposite meaning. However, most previous studies have confirmed that women are more embarrassed than men (Costa et al., 2001; Hall, 2011; Miller, 1992; Miller, 1987). The findings of this study are novel and have some implications for further theoretical research and the development of WeChat and other social networking services. According to the research results, 18–25-year-old users are more susceptible to embarrassment when missending WeChat emojis, compared with 31–40-year-old users. Previous research has pointed out that young people, aged between 18 and 25 years, often use emojis and say that these can express their emotions better (Zhang et al., 2017). As for the number of participants in this study, the majority of participants were between 18 and 25 years old. The use of emojis decreases with age (Dhir & Tsai, 2017; Ozimek et al., 2017; Zhan et al., 2016). There is a correlation between emojis with opposite meanings and the frequency of emoji usage. The use of emojis is related to age (Wang et al., 2018), as younger generations (i.e., college students) are very active users of MIMs (Oleszkiewicz et al., 2017). WeChat is the main mobile and MIM application among young Chinese people (Cohen-Zada & Krumer, 2017). People in the 18–25-year-old age group were also more likely to be embarrassed by mistakenly sending emoji with opposite meanings. These findings will also provide a reference for MIM developers.

Our research, like other studies, has its research limitations. First, it is difficult to collect samples that meet the requirements of this study. Although the number of samples meets the planned number of studies, the number of samples is still small, compared with other studies. It is important to acknowledge that our sample was small, generating smaller power in our results (Laurence et al., 2020). Secondly, when we define emoji with an opposite meaning, we mainly consider a specific meaning, context, and language culture. However, there may be other factors that affect people’s understanding of emoji with opposite meanings. Thirdly, as this study takes WeChat as an example, its scope is limited to a single language and culture background and independent instant messaging applications. Therefore, we cannot understand the difference of embarrassment caused by sending emoji with opposite meanings in other languages, cultures, and instant messaging software.

## Conclusion and suggestions

Because embarrassment has adverse consequences, eliminating it in WeChat communication can enhance the interaction between users and make them willing to continue to use the social platform. As previous scholars have studied one or several important embarrassment-related emotions, this study explored the embarrassment caused by missending emojis with opposite meanings, and it has provided several important embarrassment-related emotions. Surprisingly, we found that males in the study were more susceptible to embarrassment than females. In addition, when peers use WeChat, people are more motivated to maintain relationships (Borg et al., 1988; Kaufman, 2004; Lewis, 1971). It should be further verified in future studies whether it is possible that males are more willing to maintain social relationships than females in WeChat information interaction. Young users aged between 18 and 30 years are more susceptible to embarrassment when they mistakenly send WeChat emojis. On missending WeChat emojis with opposite meanings, the major components that explain Embarrassment are Shame, Anxious, and Upset. Reducing the users’ emotional reaction of shame can improve their willingness to continuously use WeChat. We thus encourage real-time communication app developers to make full use of these findings, to design the right emoji alignment to reduce user embarrassment, to promote the friendly social interaction of real-time communication apps, and to achieve the good sustainable development of network social interaction.

According to the research limitations, we propose, firstly, that future research should collect more samples that meet the research requirements by using effective sample collection methods. Secondly, when defining the opposite meanings of emoji, researchers should consider the influence of other aspects. For example, the meaning of emoji may be related to social geography (Scheff, 2015). Considering the influence of more factors on the opposite meaning of emoji will help to generalize the research results. Thirdly, in the future research, we should compare the differences and connections of the opposite meanings of emoji in different languages and cultural backgrounds. In addition, we should analyze the differences between the users’ understanding of the opposite meaning of emoji in different instant messaging software. Based on the above two aspects, researchers can compare different research results to obtain new conclusions.

## Figures and Tables

**Table 5:**
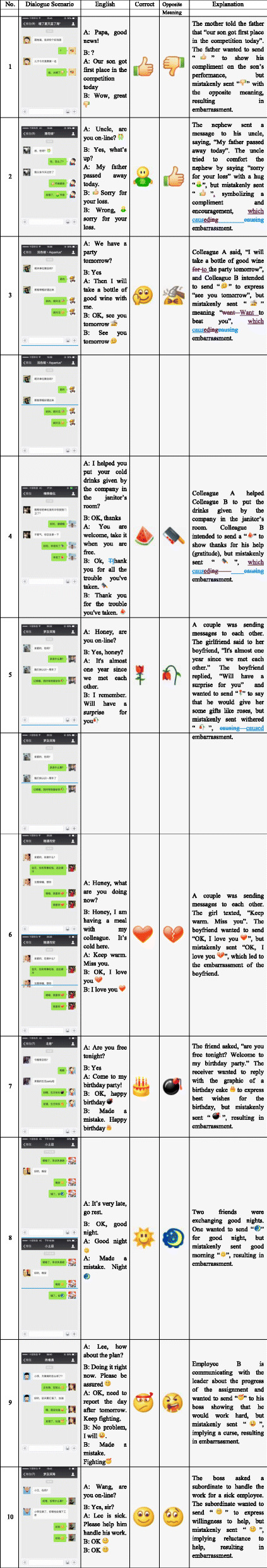
Ten dialogue scenarios and explanations

## Data Availability

The datasets used and/or analyzed during the current study are available from the corresponding author on reasonable request.
